# Ferric Chelate Reductase 1 Like Protein (FRRS1L) Associates with Dynein Vesicles and Regulates Glutamatergic Synaptic Transmission

**DOI:** 10.3389/fnmol.2017.00402

**Published:** 2017-12-08

**Authors:** Wenyan Han, Huiqing Wang, Jun Li, Shizhong Zhang, Wei Lu

**Affiliations:** ^1^Synapse and Neural Circuit Research Unit, National Institute of Neurological Disorders and Stroke, National Institutes of Health, Bethesda, MD, United States; ^2^Department of Neurosurgery, The First Affiliated Hospital of Fujian Medical University, Fuzhou, China; ^3^Department of Neurosurgery, Zhujiang Hospital, Southern Medical University, Guangzhou, China

**Keywords:** FRRS1L, AMPA receptor, C9orf4, auxiliary subunit, dynein

## Abstract

In the brain, AMPA receptors (AMPARs)-mediated excitatory synaptic transmission is critically regulated by the receptor auxiliary subunits. Recent proteomic studies have identified that Ferric Chelate Reductase 1 Like protein (FRRS1L), whose mutations in human lead to epilepsy, choreoathetosis, and cognitive deficits, is present in native AMPAR complexes in the brain. Here we have characterized FRRS1L in both heterologous cells and in mouse neurons. We found that FRRS1L interacts with both GluA1 and GluA2 subunits of AMPARs, but does not form dimers/oligomers, in HEK cells. In mouse hippocampal neurons, recombinant FRRS1L at the neuronal surface partially co-localizes with GluA1 and primarily localizes at non-synaptic membranes. In addition, native FRRS1L in hippocampus is localized at dynein, but not kinesin5B, vesicles. Functionally, over-expression of FRRS1L in hippocampal neurons does not change glutamatergic synaptic transmission. In contrast, single-cell knockout (KO) of FRRS1L strongly reduces the expression levels of the GluA1 subunit at the neuronal surface, and significantly decreases AMPAR-mediated synaptic transmission in mouse hippocampal pyramidal neurons. Taken together, these data characterize FRRS1L in heterologous cells and neurons, and reveal an important role of FRRS1L in the regulation of excitatory synaptic strength.

## Introduction

In the brain, fast excitatory synaptic transmission is largely mediated by glutamate acting on AMPA-type ionotropic glutamate receptors (AMPARs). AMPARs are heterotetrameric assemblies of four subunits, GluA1-4, and the regulation of trafficking and function of AMPARs in neurons underlies many forms of synaptic plasticity, the cellular correlate of learning and memory (Malinow and Malenka, 2002; Bredt and Nicoll, 2003; Malenka and Bear, 2004; Anggono and Huganir, 2012; Huganir and Nicoll, 2013). Accumulating studies over the last decade have shown that trafficking and function of AMPARs are critically controlled by an ever-growing list of membrane proteins, including TARPs (Chen et al., 2000; Tomita et al., 2003; Jackson and Nicoll, 2011), CNIHs (Schwenk et al., 2009), SynDig1 (Kalashnikova et al., 2010), CKAMPs/SHISAs (von Engelhardt et al., 2010; Farrow et al., 2015; Klaassen et al., 2016), GSG1L (Schwenk et al., 2012; Shanks et al., 2012; McGee et al., 2015; Gu et al., 2016a), PORCN (Erlenhardt et al., 2016), α/β-ABHD6 (Erlenhardt et al., 2016; Wei et al., 2016) and carnitine palmitoyltransferase 1C (CPT1C) (Gratacos-Batlle et al., 2014; Fado et al., 2015). These membrane proteins regulate AMPAR biogenesis, trafficking, gating, and pharmacological properties, and often play overlapping, but also distinct, roles in the regulation of AMPAR-mediated synaptic transmission (Jackson and Nicoll, 2011; Straub and Tomita, 2012; Haering et al., 2014; Greger et al., 2017; Jacobi and von Engelhardt, 2017).

Recent proteomic studies have identified several uncharacterized proteins, including transmembrane, GPI-anchored and secreted molecules, that are associated with endogenous AMPARs in the brain (von Engelhardt et al., 2010; Schwenk et al., 2012; Chen et al., 2014). Understanding the function of these molecules in the regulation of AMPARs will be important for elucidation of the regulatory mechanisms for controlling excitatory synaptic transmission. One of these proteins is FRRS1L, which is also named C9orf4 (Schwenk et al., 2012). Human and mouse genetic studies have shown a critical role of FRRS1L in human and animal behavior (Madeo et al., 2016; Shaheen et al., 2016). Indeed, loss-of-function mutations of FRRS1L in human lead to epilepsy, prominent choreoathetosis, and severe impairment of cognitive functions (Madeo et al., 2016; Shaheen et al., 2016). Thus, it is important to understand the role of FRRS1L in the regulation of synaptic transmission.

Here we have performed basic characterizations of FRRS1L in heterologous cells and in neurons, and have employed gain-of-function and loss-of-function approaches to examine the role of FRRS1L in the regulation of glutamatergic synaptic transmission. We find that over-expressed FRRS1L in hippocampal neurons only co-localizes with a portion of the AMPAR GluA1 subunit at the plasma membrane, suggesting that FRRS1L might be involved in the regulation of a subpopulation of AMPARs in neurons. Interestingly, native FRRS1L is localized at dynein, but not kinesin KIF5B, vesicles. Functionally, FRRS1L does not regulate AMPAR abundance at the cell surface in HEK cells, and similarly, over-expression of FRRS1L in culture hippocampal neurons does not change the strength of glutamatergic synaptic transmission. In contrast, single guide RNA (sgRNA)-based single-cell knockout (KO) of FRRS1L in hippocampal neurons leads to a modest, but significant, reduction of AMPAR-mediated synaptic transmission. Importantly, the sgRNA-mediated effect can be fully rescued by a sgRNA resistant FRRS1L construct in neurons. Taken together, these data reveal a distinct subcellular distribution of FRRS1L in hippocampal neurons and demonstrate an important role of FRRS1L in the regulation of AMPAR-mediated synaptic transmission in hippocampus.

## Materials and Methods

### Mouse Genetics

This study was carried out in accordance with the recommendations of the Animal Care and Use Committee (ACUC) guidelines at National Institute of Neurological Disorders and Stroke (NINDS), National Institutes of Health (NIH). The animal protocols were approved by the NINDS ACUC at NIH (protocol number: 1339-15). Mice of either sex were used. Animals were given access to food and water *ad libitum*.

### Plasmids

The plasmid encoding mouse FRRS1L was purchased from Origene (Cat. MC212878). pCAGGS/FRRS1L-IRES–EGFP plasmid was generated by inserting FRRS1L coding sequence to pCAGGS-IRES–EGFP or pCAGGS-IRES–mCherry vector. N-terminal human influenza hemagglutinin (HA) tag was inserted after the signal peptide sequence of FRRS1L (HA-FRRS1L) through an overlapping PCR strategy and the amplicon was ligated in pcDNA3.0 vector (Invitrogen). C-terminal myc tag was added through the standard PCR strategy and ligated in pcDNA3.0 vector for FRRS1L-myc plasmid. Flag-GluA1 and Flag-GluA2 (Q) plasmids were gifts from Dr. Katherine Roche’s lab at NINDS, NIH. The pspCas9(bb)-2A-IRES-GFP plasmid was purchased from Addgene (Cat. 48138) for sgRNA cloning. FRRS1L sgRNAs were generated by a general PCR procedure with the following sgRNA sequences (accession number: NP_001136437, #1: gcgggatccgtgcgcagcgatgg; #2: ccccgcggacgacagcgcgggcc; #3: accccggggacgcgcgcgagggg). FRRS1L sgRNAs were inserted into pspCas9(bb)-2A-IRES-GFP vector at the BbsI cutting site. The underlined sequence of sgRNA^#3^ was the mutation region for the sgRNA^#3^ resistant plasmid, FRRS1L^∗^ (mutation region: accccggggacgcgcgcgagggg to acccagaggcagagcaagagggg; amino acids: *Arg-Gly-Arg-Ala-Arg*) (template: FRRS1L-myc in pcDNA3.0 vector), which were inserted into pCAGGS-IRES-mCherry vector. All plasmids were confirmed by standard DNA sequencing.

### Co-immunoprecipitation and Western Blot in HEK Cells

For co-immunoprecipitation (Co-IP) experiments, Flag-GluA1, Flag-GluA2 and HA-FRRS1L constructs were used for transfection in HEK cells. HEK cells were cultured and transfected with effectene transfection reagent (Qiagen, Cat. 301425) as described previously (Gu et al., 2016a). Forty-eight hours after transfection, cells were homogenized in ice-cold lysis buffer containing 25 mM Tris (pH 7.4), 1% Triton X-100, 150 mM NaCl, 5% glycerol, 1 mM EDTA, and EDTA-free protease inhibitors (Roche, 5892791001). Equal amounts of cell lysates were incubated with anti-Flag M2 affinity beads (Sigma, A2220) overnight at 4°C. Beads were washed three times with 300 μl lysis buffer and diluted in equal amount of 2 × loading buffer (Bio-Rad 161–0737) containing 10% β-mercaptoethanol (BME) (Fisher Scientific BP176100). Samples were then resolved by SDS–PAGE with 10% precast gel (Bio-Rad 4561083) and were subjected to immunoblotting with anti-HA (rabbit, 1:1,000, Santa Cruz, sc-805) or anti-Flag (rabbit, 1:1,000, Sigma F2555) antibodies. For the dimerization experiment, HA-FRRS1L and FRRS1L-Myc plasmids were co-transfected into HEK cells for 48 h. Anti-HA and anti-Myc (rabbit, 1:1,000, Cell signaling, 2278S) antibodies were used in this experiment. For FRRS1L sgRNA screening experiment, FRRS1L-Myc and sgRNA candidates were co-transfected in a ratio at 1:2 to HEK cells (2 × 10^6^ cells/well on transfection day in 6-well plate), while empty pcDNA3.0 vector was added in FRRS1L-Myc transfected cells to balance the total amount of DNA. All data were collected from at least three independent experiments.

### Mouse Tissue Preparations and Western Blotting

Mouse tissues of interest (6–8-week old for tissue distribution experiments) were quickly dissected after anesthetizing the donor mice with isoflurane (Baxter, NDC10019-360-40), then placed in 2.0 ml microfuge tubes and kept in dry ice to snap freeze. The samples were separately added into an ice-cold glass pestle containing 1 ml ice-cold non-detergent homogenization buffer [250 mM sucrose, 1 mM EDTA, 10 mM Tris-HCl buffer (pH 7.2), and EDTA-free protease inhibitors (1 tablet/10 ml, Roche, 5892791001)] to homogenize. The homogenate was then sonicated with two 10-s pulses (30 s pause in between pulses) using an ice-cold ultrasonic probe, and then centrifuged at 1,000 × *g* for 10 min to remove neuronal nuclei and other cell debris. The crude post-nuclear supernatant fraction (PNS) was transferred to a 1.5 ml ultracentrifuge tube; the pellet containing the nuclei was discarded. The PNS was centrifuged at 100,000 × *g* for 2 h at 4°C to collect membrane fractions in the pellet. The membrane fractions were lysed in appropriate volume of ice-cold lysis buffer containing 25 mM Tris (pH 7.4), 1% Triton X-100, 150 mM NaCl, 5% glycerol, 1 mM EDTA, and EDTA-free protease inhibitors. Equal amounts of lysates measured by BCA protein assay kit (Thermofisher, Cat. 23225) were subjected to SDS-PAGE and immunoblotting with an anti-FRRS1L (1:1,000, Santa Cruz, sc-398692) or anti-α-tubulin antibody (1:5000, Sigma, T8203). All data were collected from at least three independent experiments.

### Vesicle Immunoisolation

Adult WT mouse hippocampal tissue (6–8-week old) was homogenized in ice-cold 500 μl (20 mg tissue/100 μl) detergent-free buffer (25 mM sucrose, 20 mM pH7.4 Tris-HCl), centrifuged at 1,000 × *g* for 10 min at 4°C (Encalada et al., 2011). The buffer contained an EDTA-free protease inhibitor (Roche, 5892791001). The 400 μl hippocampal homogenate was bottom-loaded on a sucrose step gradient consisting of 35% (400 μl), and 8% (600 μl) sucrose in each 1.5 ml centrifuge tube (VMR 16466-064). After centrifugation at 200,000 × g for 2 h at 4°C, the 8/35, 35/40, and post-ultracentrifuge PNS membrane interphases (PNSM) were separately harvested and incubated with Dynein (1:300, MAB1618, Millipore), Kinesin (1:300, MAB1614, Millipore) or IgG (1:300, Sigma) antibodies overnight. The next day, washed 50 μl protein G dynabeads (ThermoFisher, 10007D) were added into the antibody-incubated fractions followed by incubating with rotation for 1hr at RT. Washed pellets on magnet were eluted with 20 μl elution buffer. Eluted sample was mixed with 2× loading buffer containing 10% BME and heated at 60°C for 10 min. The 8/35, 35/40 and post-ultracentrifuge PNSM were analyzed by SDS-PAGE and immunoblotted by anti- FRRS1L (1:1,000), γ8 (1:1,000, rabbit, SigmaMillipore, Cat. AB9876) or CNIH2 (1:1,000, rabbit, Synaptic Systems, Cat. 253203) antibodies. The 8/35 interphase incubated with dynein/kinesin was also immunoblotted by anti-AMPAR_(PAN)_ (1:1,000, mouse, SigmaMillipore, MABN832) and dynein/kinesin antibodies.

### Immunocytochemistry in HEK Cells

For surface and total AMPAR subunit expression, HEK cells (2 × 10^4^ cells/coverslip) cultured on coverslips were transfected with Flag-GluA1 or Flag-GluA2 on their own or together with pCAGGS/FRRS1L-IRES-mCherry by effectene transfection reagent, and incubated in 37°C incubator containing 5% CO_2_ for approximate 40–48 h. After removing the cell media, cells were washed once by 1xPBS and then fixed with ice-cold fixation buffer (10 ml 16% paraformaldehyde solution, 8 ml 5× PBS and 1.6 g sucrose dissolved in 22 ml distilled water) for 15 min. Cells were then blocked by PBS containing 10% normal goat serum (NGS) (Vector Laboratories) for 30 min. Without permeabilization, cells were incubated with a monoclonal anti-Flag antibody (1:1,000) dissolved in 1× PBS containing 3% NGS at room temperature (RT) for 2 h for surface Flag (sFlag) tag labeling. After three times washing by 1x PBS, cells were permeabilized with 0.2% Triton X-100 for 15 min, and then blocked for 30 min in 1× PBS containing 10% NGS. Cells were then incubated with a polyclonal anti-Flag antibody (1:1,000) at 4°C overnight for total Flag (tFlag) tag labeling. Alexa Fluor (AF) 647 anti-mouse and AF488 anti-rabbit secondary antibodies (Molecular Probe) were used to label sFlag and tFlag. For co-localization of surface and total HA-FRRS1L or sFlag- GluA1/GluA2 in HEK cells, the cells were transfected and treated as above with indicated plasmids, and were subjected to similar immunolabeling procedures as described above. Coverslips were mounted with Fluoromount G (Southern Biotech) for imaging acquisition.

### Hippocampal Primary Dissociated Neuronal Culture

Hippocampal primary neuron cultures were prepared as described before (Gu et al., 2016b). Briefly, hippocampi from ∼E18 C57B6 mouse embryos were microdissected in ice-cold dissection media (49 ml Ca^2+^/Mg^2+^ free Hanks media, 0.5 ml 1M HEPEs and 0.5 ml 100x Pen/strep) and incubated in papain solution (papain (110 u/vial) dissolved in 5 ml Ca^2+^/Mg^2+^ free Hanks media and 250 μl DNase-I (1100 u/vial) for 45 min followed by centrifuging at 800 rpm for 5 min. The pellet was re-suspended in Hanks solution (Invitrogen 14025-092) containing DNase-I (Worthington 3170) and dissociated into single cells by gentle pipette trituration. Digestion was then stopped by adding trypsin inhibitor (10 mg/ml, Sigma T9253) and BSA (10 mg/ml, Sigma A9647), and centrifuged at 800 rpm for 10 min. The pellet was re-suspended in neurobasal plating media containing 2% FBS, 2% B27 supplements, and L-glutamine (2 mM). Neurons were plated at a density of ∼1.5 × 10^5^ cells/coverslip on coverslips pretreated with poly-D-lysine (Sigma P7886) in a 24-well plate. Culture media were replaced by half once per week.

### Immunocytochemistry in Neurons

Mouse hippocampal cultured neurons were transfected with indicated FRRS1L sgRNA^#1^ or sgRNA^#3^ plasmids by calcium phosphate transfection at DIV2 as described (Li et al., 2017). Endogenous GluA1 in live neurons was labeled with a mouse monoclonal antibody against an extracellular epitope of GluA1 (clone RH95; 5 μg/ml) in conditioned culture medium for 15 min at 37°C at DIV16. After being washed by 1× PBS, neurons were fixed with ice-cold fixation buffer for 15 min. After permeabilization using 0.2% Triton X-100 in 1× PBS for 15 min at RT, neurons were blocked for 1 h in 1× PBS containing 5% NGS. Cells were then incubated with anti-GluA1 (SigmaMillipore, AB1504, 1:1,000) antibody at 4°C overnight followed by incubation with AF-647 (tGluA1) and AF-555 secondary antibodies (sGluA1, Molecular Probe). In FRRS1L overexpression experiments, mouse hippocampal dissociated neurons were transfected with HA-FRRS1L plasmid by DNA-In neuro transfection reagent (MTI-GlobalStem, Cat. GST-2101) at DIV10. Cells at DIV14-16 were immunostained as described above. For co-localization of surface HA-FRRS1L (sHA-FRRS1L) and surface endogenous GluA1, GluA2 or PSD95 (anti-PSD95, Sigma Millipore, AB9708), neurons were transfected with HA-FRRS1L by Neuro-In DNA transfection reagent at DIV10 and fixed on DIV16.

### Image Acquisition

For immunostaining in HEK cells, images were acquired on a Zeiss LSM 880 laser scanning confocal microscope using a 63x oil objective (1.4 numerical aperture). Multiple *z* sections (9 optical slices) were acquired at 0.5–1.0 μm intervals. Images were captured using a 1024 × 1024 pixel screen, and gains for both fluorophores were set between 700 and 800. For immunostaining in neuronal cultures, images were acquired on a Zeiss LSM 880 laser scanning confocal microscope using a 63x oil objective (1.4 numerical aperture). Image acquisition was performed with identical settings for a specific experiment. Multiple z sections of secondary apical dendrites were collected at 1.0–1.5 μm intervals with 512 × 512 pixel screen. Pinhole was set to 1 airy unit for all experiments. Scan speed function was set to 9 and the mean of 4 lines was applied. Laser power, digital gain, and offset settings were all identical in each experiment by using the “reuse” function in LSM software.

### Images Analysis

For quantitative analysis of immunostaining in HEK cells and in neurons, maximal projection images were created with ZEN software (Zeiss) from 4 to 6 serial optical sections. For each image collected containing sGluA1 or PSD95, dendritic outline was drawn to cover 20–30 μm in length (representing a surface area of 850–1,000 pixels). The integrated fluorescent intensity targeting protein was calculated from one segment of dendrite positive for HA-FRRS1L. Using Metamorph (Universal Imaging Corp.), quantitation of the fluorescence signal from sFlag or tFlag in HEK cells and surface/total AMPAR subunits in neurons were determined from fluorescent signal above a threshold. The threshold value was held identical within single experiments, and only slightly adjusted between independent experiments. For surface/total ratio of GluA1 staining and GFP fluorescent signals in dissociated hippocampal neuronal cultures, maximal projection images were generated by the LSM880 Brower software. Background was subtracted by using the “subtract background” function in ImageJ software (NIH), and the background level was held identical for all cells within each experiment. Region of interest (ROI) was defined along a segment of the dendrite 20–30 μm according to the fluorescence signal distinguished from the background in ImageJ software. Average values of fluorescence intensities in ROI (the total fluorescent intensity divided by the total area of a dendritic segment) were calculated by ImageJ.

For co-localization analysis in cultured hippocampal neurons, 25–30 μm secondary dendritic segments were chosen as ROI. HA-FRRS1L, GluA1 or PSD-95 puncta were thresholded and confirmed visually to select appropriate clusters following a minimal size cut-off, which included all recognizable clusters. The co-localization percentage was quantified by the measurement of HA-FRRS1L-positive GluA1 or PSD-95 puncta divided by total number of thresholded GluA1 or PSD-95 puncta. 10–27 dendrites were analyzed from approximately 5–10 neurons.

### *In Situ* Hybridization (ISH) in Cultured Neurons

Cultured neurons on coverslips were transfected with sgRNAs at DIV2 and fixed in fixation buffer at RT for 15 min at DIV16. Coverslips were then washed three times with 1× PBS and sequentially incubated in 50, 70, and 100% EtOH for 5 min for dehydration. The coverslips were then submerged in 70 and 50% EtOH for 2 min for rehydration followed by keeping in 1× PBS for 10 min. The coverslips were then treated with hydrogen peroxide and protease III (1:15 diluted in 1× PBS) (RNAscope, 2000258) for 10 min followed by washing twice in 1× PBS. The coverslips were quickly transferred to the barriered SuperFrost Plus slide. All of the above steps were operated at RT. Four drops (∼50–80 μl) of FRRS1L probe (RNAscope, 16137A) were added to submerge the coverslip, then the slide was incubated in a humidity control tray at 40°C for 2 h followed by washing twice in 1× wash buffer (RNAscope, 2000003) for 2 min at RT. The slide was then respectively incubated with detection reagents AMP1-6 and Fast RED reagent (A:B, 1:60 ratio) (RNAscope, 2000173) as described in RNAscope HD 2.5 RED reagent protocol (322360-USM). After rinsing in 1x wash buffer, the slide was removed from the humidity control tray and dried in dry oven at 60°C for 20 min. The slide containing coverslip was then dipped in fresh Xylene and 3 drops of DAPI permount media were quickly added followed by covering with a 24 mm × 50 mm coverslip without air bubbles.

Positive punctate signal was detected under a standard bright field microscope at 63x magnification using confocal microscope. GFP positive signals and red punctate dots were presented in FITC and 550 nm channels. The staining of ISH was categorized into five grades based on the recommendation from the RNAscope manufacturer (322360-USM): 0, 1+, 2+, 3+, and 4+. Staining Scores were defined by the following criteria: 0, no staining or less than 1 dot per 10 cells; 1, 1–3 dots per cell; 2, 4–10 dots per cell, very few dot clusters; 3, >10 dots per cell, less than 10% positive cells have dot clusters; 4, >10 dots per cell, more than 10% positive cells have dot clusters.

### *In Utero* Electroporation

Timed-pregnant WT C57B6 mice at embryonic 14.5 days (E14.5) were anesthetized with isoflurane. The abdominal cavity was opened and 7–9 embryos in the uterine horns were gently exposed. The lateral ventricle of each embryo was manually injected with approximately 1–2 μl of FRRS1L sgRNA plasmid at a concentration of 2 μg/μl mixed with 0.05% fast green. The pipettes were beveled with a BV-10 micropipette Beveller (Sutter) before microinjection. After unilateral injection in embryos, voltage steps via tweezertrodes (5 mm round, platinum electrodes and BTX electroporator, BTX, ECM830) positioned on either side of the embryo head were applied across the uterus to target hippocampal neural progenitors. Voltage was set at 45 V for 5 pulses at 1 Hz, each pulse lasting 50 ms. The embryos were then moistened with sterilized PBS (pre-warmed at 37°C) and returned to the abdominal cavity. Buprenex (0.1 mg/kg) was then applied and the wound was subsequently sutured.

### Electrophysiology in Hippocampal Slices

Organotypic hippocampal slice cultures were prepared and transfected as previously described (Gu et al., 2016a). Both sexes of C57B6 mice at the age of p6–p8 were used for organotypic hippocampal slice cultures. About 2–4 days after culture, slices were transfected with FRRS1L-IRES-EGFP or with FRRS1L sgRNA^#1^ or sgRNA^#3^ plasmids by gene gun-mediated transfection. Slices were cultured for an additional 2–5 days for over-expression or 14–16 days for sgRNA transfection. After transfection, slices were transferred to a submersion chamber on an upright Olympus microscope, perfused in normal ACSF containing (in mM) NaCl 119, KCl 2.5, NaHCO_3_ 26.2, NaH_2_PO_4_ 1, glucose 11, CaCl_2_ 4.0, and MgSO_4_ 4.0, with picrotoxin (100 μM), and saturated with 95% O_2_/5% CO_2_. For recording evoked EPSCs in organotypic slices, ACSF was also supplemented with 5–20 μM 2-chloroadenosine to dampen epileptiform activity. EGFP-positive neurons in organotypic slice cultures were identified by epifluorescence microscopy. For acute hippocampal slices, mouse pups at p14–p16 after embryonic IUE were euthanized by decapitation. The brain was immediately placed in ice-cold cutting solution containing (in mM) KCl 2.5, CaCl_2_ 0.5, MgCl_2_ 7, NaH_2_PO_4_ 1.25, NaHCO_3_ 25, glucose 7, ascorbic acid 1.3 and sucrose 210 as previously described (Gu et al., 2016b). The hippocampi were quickly dissected out on an ice-cold platform and immediately placed onto an ice-cold Argrose gel block (5% Argrose). The gel block was quickly glued on the cutting platform containing ice-cold cutting solution saturated with carbogen. 300 μm transverse slices were cut and recovered at 32°C for 30–60 min. Slices were then maintained in ACSF (modified to containing 2.5 mM CaCl_2_ and 1.3 mM MgCl_2_) at RT for 30–60 min prior to recording. The slice was then transferred to a recording chamber that is mounted on an upright Olympus microscope. The recording chamber was continuously perfused with ACSF containing picrotoxin (100 μM) and saturated with 95% O_2_/5% CO_2_.

All paired recordings involved simultaneous whole cell recordings from one EGFP-positive neuron and a neighboring EGFP-negative control neuron. Cells were recorded with 3- to 5- MO borosilicate glass pipettes. The internal solution contained CsMeSO_4_ 135 mM, NaCl 8 mM, HEPEs 10 mM, Na_3_GTP 0.3 mM, MgATP 4 mM, EGTA 0.3 mM, QX-314 5 mM and spermine 0.1 mM. The stimulus was adjusted to evoke a measurable, monosynaptic EPSC in both cells. AMPA EPSCs were measured at a holding potential of -70 mV, and NMDA EPSCs were measured at +40 mV and at 100 ms after the stimulus, at which point the AMPA EPSC has completely decayed. Paired-pulse ratios (PPRs) were measured by giving two pulses at a 50-ms interval and taking the ratio of the two peaks of the EPSCs from an average of 30–50 sweeps. Series resistance was monitored and not compensated, and cells in which series resistance varied by 25% during a recording session were discarded. Synaptic responses were collected with a Multiclamp 700B amplifier (Axon Instruments, Foster City, CA, United States), filtered at 2 kHz and digitized at 10 kHz. All pharmacological reagents were purchased from Abcam, and other chemicals were purchased from Sigma.

In the scatter plots for simultaneous dual recordings (**Figures 4, 6**), each open circle represents one paired recording, and the closed circle represents the average of all paired recordings. In the scatter plot, the x-axis represents the EPSC recorded in the control cell, and the y-axis represents the EPSC recorded in the transfected cell. Virtual 1:1 diagonal line is also shown. If the data point falls above the diagonal line, it indicates that the EPSC is higher in the transfected cell. If it falls below the diagonal line, it indicates that the EPSC is higher in the control cell.

For decay time constants of AMPA EPSCs, a single weighted decay time constant was calculated from the area under the peak-normalized current (Cathala et al., 2005), according to

$$\begin{array}{c} t_{\mathrm{decay}}=\frac{1}{l_{\mathrm{peak}}}\int_{t_{\mathrm{peak}}}^{t_{0}}l(t)\mathrm{dt}, \end{array}$$

where *t*_0_ was 80 ms after the peak.

### Glutamate Puffing Experiments in HEK Cells

For glutamate-evoked whole-cell currents in HEK cells, cells (2 × 10^4^ cells/coverslip) were cultured and transfected with GluA1, GluA2, GluA1 plus FRRS1L-IRES-GFP (ratio at 1:1), or GluA2 plus FRRS1L-IRES-GFP (ratio at 1:1) (an empty plasmid was used to make the total cDNA amount, 0.4 μg, per transfection). On the day of recording, coverslips with transfected HEK cells were transferred to a submersion chamber on an upright Olympus microscope perfused with external solution containing (in mM) NaCl 140, KCl 5, MgCl_2_ 1.4, EGTA 5, HEPES 10, NaH_2_PO_4_ 1, D-glucose 10, and NBQX 0.01, with pH adjusted to 7.4. The internal solution contained CsMeSO_4_ 135 mM, NaCl 8 mM, HEPEs 10 mM, Na_3_GTP 0.3 mM, MgATP 4 mM, EGTA 0.3 mM, QX-314 5 mM, and spermine 0.1 mM. Glutamate induced whole-cell currents in HEK cells were recorded at -70 mV by local fast application (0.5 s) of 1 mM glutamate and 100 μM cyclothiazide, dissolved in the external solution. The tip of Mini-manifold was placed at 100 μm away from the recorded HEK cells. Whole-cell currents were collected with a Multiclamp 700B amplifier (Axon Instruments, Foster City, CA, United States), filtered at 2 kHz and digitized at 10 kHz. All pharmacological reagents were purchased from Abcam, and other chemicals were purchased from Sigma.

### Statistical Analysis

Statistical analysis was performed using GraphPad Prism 7. Comparisons between two groups were performed with two-tailed, unpaired *t*-tests except for paired recording whereby the statistic comparisons were performed with two-tailed, paired *t*-test. Multiple comparisons with three or more groups were performed with one-way ANOVAs. The D’Agostino and Pearson normality test and the Shapiro–Wilk normality test were performed to test the normality of unpaired data. All of the data passed the normality test (α = 0.05) and came from a Gaussian distribution (*p* > 0.05). Significance was considered when the *p*-value < 0.05, 0.01, 0.001 or 0.0001 (indicated as ^∗^, ^∗∗^, ^∗∗∗^, or ^∗∗∗∗^, respectively). *p*-values ≥ 0.05 were considered not significant. The data were presented as Mean ± SEM.

## Results

### Basic Characterizations of FRRS1L in Mouse

**FIGURE 1:**
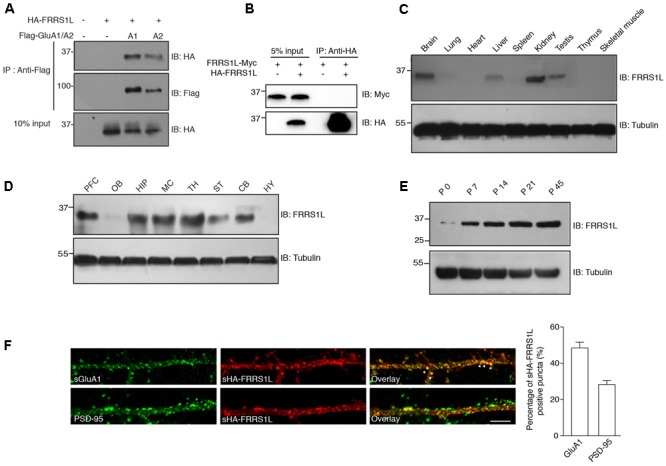
Basic characterization of FRRS1L protein in mice. **(A)** Co-IP of FRRS1L with GluA1 or GluA2(Q) in HEK cells. Lysates from HEK cells transfected with HA-FRRS1L alone, HA-FRRS1L together with Flag- GluA1 or GluA2(Q), or an empty vector as a control, were immunoprecipitated with an anti-Flag antibody. The IPs and 10% input were probed with indicated antibodies. IB, immunoblotting. *N* = 3 independent repeats. **(B)** FRRS1L did not form dimers or oligomers in HEK cells. Lysates from HEK cells transfected with FRRS1L-Myc alone or together with HA-FRRS1L were immunoprecipitated with an anti-HA antibody. The IPs and 5% input were probed with indicated antibodies. *N* = 3 independent repeats. **(C)** Tissue distribution of endogenous FRRS1L in adult mouse. *N* = 4, *n* = 5 animals. **(D)** Expression profile of FRRS1L in adult mouse brain tissues. PFC, prefrontal cortex; OB, olfactory bulb; HIP, hippocampus; MC, motor cortex; TH, thalamus; ST, striatum; CB, cerebellum; HY, hypothalamus. *N* = 3 independent repeats from 6 animals. **(E)** Developmental profile of FRRS1L expression in mouse hippocampus. *N* = 4 independent repeats. **(F)** Representative images show that surface HA-FRRS1L (sHA-FRRS1L) in mouse dissociated hippocampal neuron cultures at DIV16 partially co-localizes with surface GluA1 (sGluA1), but the majority of sHA-FRRS1L does not co-localize with an excitatory synapse marker, PSD-95. Arrowheads indicate the co-localization of sHA-FRRS1L with sGluA1. The bar graph in right shows the percentage of co-localization. Scale bar, 20 μm. *N* = 3 independent repeats. Uncropped scans of Western blots in Supplementary Figures 1A–E.

Recent proteomic studies have identified FRRS1L as a component of native AMPAR complexes in the brain (Schwenk et al., 2012; Chen et al., 2014). To confirm FRRS1L-AMPAR association, we performed a co-immunoprecipitation (Co-IP) assay in human embryonic kidney 293T (HEK) cells. We found that HA-tagged FRRS1L (HA-FRRS1L) could be co-immunoprecipitated with Flag-tagged GluA1 (Flag-GluA1) or Flag-tagged GluA2 (Flag-GluA2) subunit of the AMPAR from cells co-transfected with both constructs, but not from control cells transfected with either one (**Figure 1A**), indicating that FRRS1L can associate with both GluA1 and GluA2 in HEK cells. In addition, we found that in heterologous cells, FRRS1L did not form dimers or oligomers, as Myc-tagged FRRS1L (FRRS1L-Myc) was not co-immunoprecipitated by HA-FRRS1L in HEK cells (**Figure 1B**).

In mouse, FRRS1L protein was detected in the brain as well as in a few other tissues (**Figure 1C**). In the brain, FRRS1L had a broad distribution with high expression observed in hippocampus, cortex and thalamus (**Figure 1D**). In addition, expression of FRRS1L in the brain steadily increased during the development with high expression levels reached at ∼postnatal two- to three-week-old (**Figure 1E**). In dissociated hippocampal primary neuron cultures, the majority of neuronal surface HA-FRRS1L (sHA-FRRS1L) did not co-localize with an excitatory synapse marker, PSD-95 (**Figure 1F**), suggesting that FRRS1L may mainly exert its function outside of synapses. In addition, a substantial fraction of sHA-FRRS1L co-localized with a portion of endogenous surface GluA1 (sGluA1) (**Figure 1F**), indicating that FRRS1L associates with a subpopulation of GluA1-containing AMPARs in hippocampal neurons.

### FRRS1L Is Localized at Dynein-Positive Vesicles

**FIGURE 2:**
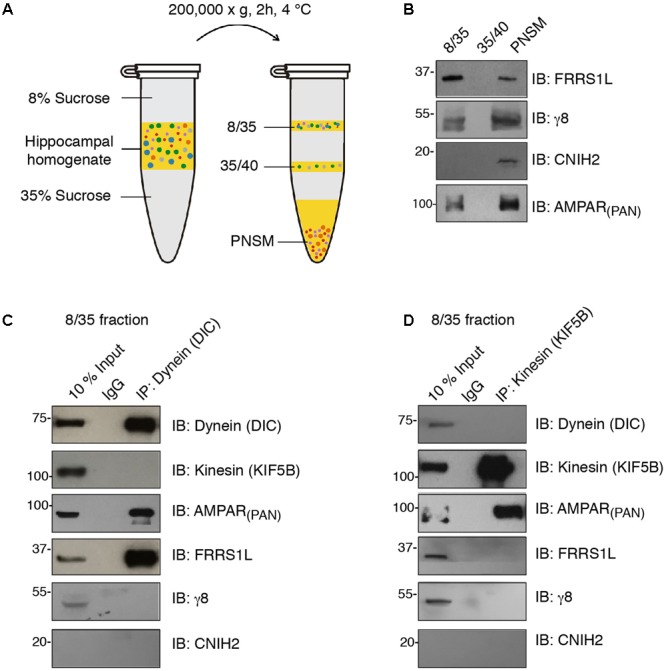
FRRS1L is localized at dynein, but not kinesin, vesicles in hippocampus. **(A)** The schematic of vesicle membrane preparation from detergent-free hippocampal homogenate. **(B)** Differential distributions of FRRS1L, γ8, CNIH2, and the AMPAR (probed by a pan-AMPAR antibody) in 8/35, 35/40, and post-ultracentrifugation PNS membrane fractions (PNSM) (*N* = 4 independent repeats). **(C,D)** Vesicle immunoisolation of 8/35 membrane fraction by a dynein (**C**, the antibody was against the dynein intermediate chain, DIC) or kinesin (**D**, the antibody was against the KIF5B) antibodies. The 10% input was also included. The immunoprecipitates were mixed with SDS-PAGE sample buffer and subjected to Western blotting by indicated antibodies (*N* = 5 independent repeats). Uncropped scans of Western blots in Supplementary Figures 2B–D.

Microtubule-based transport by dynein and kinesin motors has been shown to play an important role in the regulation of AMPAR trafficking (Kim and Lisman, 2001; Setou et al., 2002; Kapitein et al., 2010; Hoerndli et al., 2013, 2015; Heisler et al., 2014). We were wondering whether FRRS1L shows any specificity to dynein or kinesin vesicles. To this end, we performed immunoisolation of vesicles in floated membrane fractions isolated from detergent-free hippocampal homogenate with sucrose step-gradients and utilized Western blotting to analyze the proteins associated biochemically with the vesicles (**Figure 2A**). We found that AMPARs, FRRS1L, TARP γ8 and CNIH2 were detected in the PNSM, but not in the 35/40 fraction, suggesting that AMPARs and auxiliary subunits are not associated with the specific 35/40 membrane fraction (**Figure 2B**). Interestingly, in the vesicle-enriched 8/35 fraction (Encalada et al., 2011), while AMPARs, FRRS1L, and TARP γ8 were detected, CNIH2 was not detectable, indicating a diverse distribution of AMPAR auxiliary subunits in distinct membranous entities (**Figure 2B**).

In the 8/35 membrane fraction, an antibody against dynein intermediate chain (DIC) pulled down dynein, but not one of the major kinesin heavy chains in neurons, KIF5B (**Figure 2C**). Significantly, we found AMPARs and FRRS1L, but not TARP γ8 and CNIH2, in dynein vesicles, confirming an association of endogenous FRRS1L with dynein vesicles (**Figure 2C**). Interestingly, an antibody against KIF5B pulled down KIF5B and AMPARs, but none of three auxiliary subunits of AMPARs: FRRS1L, TARP γ8, and CNIH2 (**Figure 2D**), suggesting that AMPARs in KIF5B vesicles unlikely contain FRRS1L, TARP γ8 or CNIH2. However, we could not exclude the possibility that the levels of these auxiliary subunits in KIF5B immunoprecipitates in the 8/35 membrane fraction may be below the detection thresholds of corresponding antibodies. Taken together, these biochemical data demonstrate that a portion of native FRRS1L associates with dynein vesicles. These data also indicate the existence of distinct subpopulations of hippocampal AMPARs that are decorated with unique combinations of auxiliary subunits.

### FRRS1L Does Not Change the Expression of AMPARs at the Plasma Membrane in HEK Cells

**FIGURE 3:**
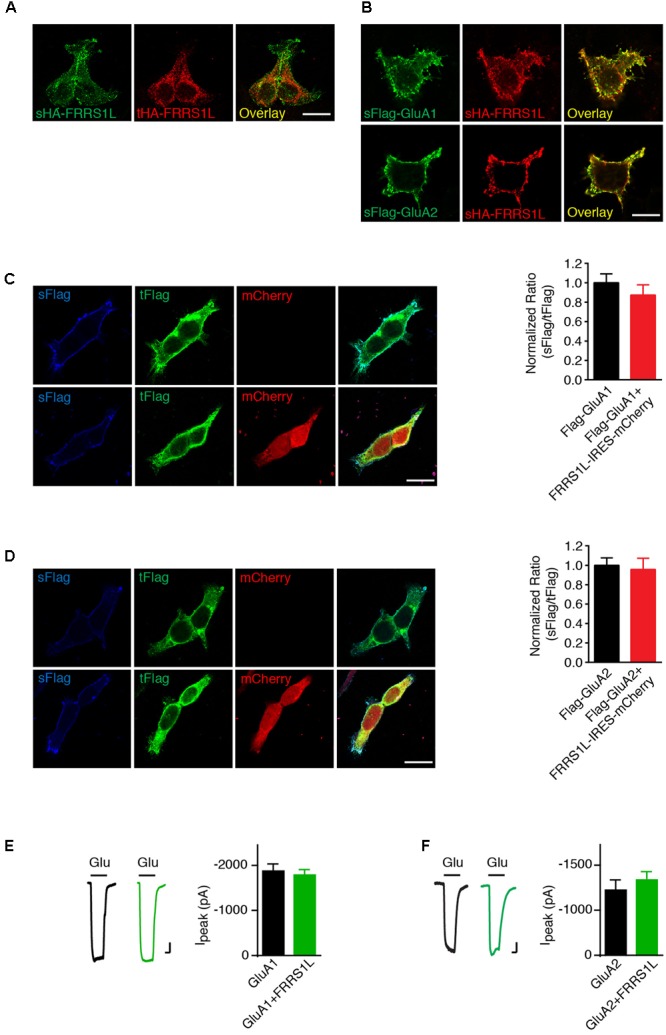
FRRS1L does not change the expression of AMPARs at the plasma membrane in HEK cells. **(A)** Representative images of surface and total HA-FRRS1L show that FRRS1L was expressed at the HEK cell surface. Scale bar, 20 μm. **(B)** Representative images of sHA-FRRS1L and sFlag-GluA1 (top) or sFlag-GluA2(Q) (bottom) show that sHA-FRRS1L co-localized with sFlag-GluA1 or sFlag-GluA2. Scale bar, 20 μm. **(C,D)** Representative images of HEK cells in left panels show that FRRS1L did not change the ratio of surface and total AMPAR expression. HEK cells were transfected with Flag-GluA1 **(C)** or Flag-GluA2(Q) **(D)** on its own, or together with FRRS1L-IRES-mCherry. Bar graphs at right show normalized ratio of surface to total GluA1 (Flag-GluA1: *n* = 15; Flag-GluA1 + FRRS1L-IRES-mCherry: *n* = 21, *p* = 0.40, *t*-test) or GluA2 (Flag-GluA2: *n* = 15; Flag-GluA2 + FRRS1L-IRES-mCherry: *n* = 20, *p* = 0.77, *t*-test) expression. sFlag, surface Flag; tFlag, total Flag. Scale bar, 20 μm. **(E,F)** FRRS1L did not affect GluA1- (**E**, GluA1: *n* = 18; GluA1 + FRRS1L: *n* = 25, *p* = 0.65, *t*-test) or GluA2(Q)- (**F**, GluA2: *n* = 17; GluA2 + FRRS1L: *n* = 25, *p* = 0.82, *t*-test) mediated, 1 mM glutamate-evoked whole-cell currents in the presence of cyclothiazide in HEK cells. Glu, glutamate. Scale bar, 200 pA and 500 ms.

We then sought to determine the role of FRRS1L in the regulation of AMPARs. Membrane proteins that interact with AMPARs often regulate AMPAR trafficking (Jackson and Nicoll, 2011; Straub and Tomita, 2012). To study FRRS1L-mediated effect on AMPAR trafficking, we first expressed AMPAR subunits on their own or together with FRRS1L in HEK cells and employed an immunocytochemical assay to examine the ratio of surface to total expression levels of AMPAR subunits. In HEK cells, HA-FRRS1L on its own could traffic to the cell surface (**Figure 3A**). In addition, sHA-FRRS1L co-localized with surface Flag-GluA1 (sFlag-GluA1) or surface Flag-GluA2 (Q) (sFlag-GluA2) in HEK cells (**Figure 3B**), indicating that FRRS1L is associated with AMPARs on the cell surface. Furthermore, we found that levels of sFlag-GluA1 or sFlag-GluA2 in HEK cells were not altered by co-expression with FRRS1L-IRES-mCherry (**Figures 3C,D**). We also co-expressed FRRS1L-IRES-mCherry together with GluA1-IRES-EGFP or GluA2-IRES-EGFP in HEK cells and conducted patch clamp recordings to measure AMPAR-mediated whole-cell currents. We found that the AMPAR subunit expressed on its own generated the same amount of whole-cell currents evoked by 1 mM glutamate (in the presence of cyclothiazide to block receptor desensitization) as the AMPAR subunit and FRRS1L expressed together (**Figures 3E,F**). Taken together, these data indicate that the abundance of surface AMPAR subunits is not changed by FRRS1L co-expression in HEK cells.

### Over-Expression of FRRS1L Does Not Affect AMPAR-Mediated Synaptic Transmission in Hippocampal CA1 Neurons

**FIGURE 4:**
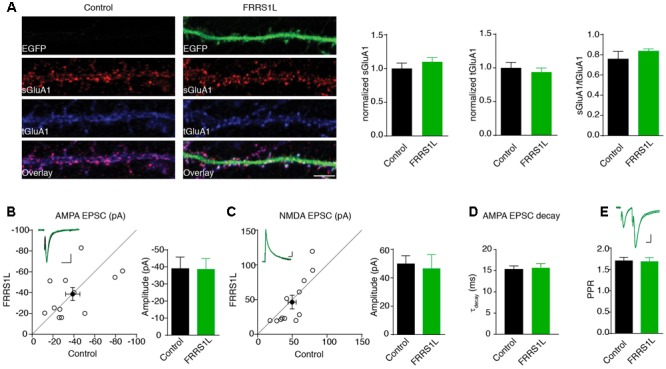
Over-expression of FRRS1L does not change AMPA receptor-mediated synaptic transmission. **(A)** Representative image of dendrites shows that over-expression of FRRS1L did not change GluA1 surface and total levels nor the ratio of GluA1 surface to total expression in cultured hippocampal neurons (Control: *n* = 12; FRRS1L: *n* = 26, sGluA1: *p* = 0.36; tGluA1: *p* = 0.57; sGluA1/tGluA1: *p* = 0.21, *t*-test). **(B,C)** Over-expression of FRRS1L did not change AMPA (**B**, *n* = 12, *p* = 0.96, paired *t*-test) and NMDA (**C**, *n* = 12, *p* = 0.10, paired *t*-test) EPSCs of hippocampal pyramidal neurons in organotypic hippocampal slice cultures. The open circles represent all pair recordings, the solid circle shows the average of all pair recordings. Scale bar, 20 pA and 100 ms for AMPA EPSC; 20 pA and 40 ms for NMDA EPSC. **(D)** The weighted decay time constant of AMPA EPSCs was not changed in neurons over-expressing FRRS1L (*n* = 12 for both conditions, *p* = 0.46, *t*-test). **(E)** The paired pulse ratio (PPR) was not changed in neurons over-expressing FRRS1L (*n* = 12, *p* = 0.81, *t*-test). Scale bar, 20 pA and 100 ms.

To study the role of FRRS1L in neurons, we first performed an immunocytochemical assay to measure surface and total levels of GluA1, a major AMPAR subunit expressed in hippocampal neurons (Lu et al., 2009), in dissociated primary hippocampal cultures that were transfected with FRRS1L-IRES-EGFP. Double immunolabeling of surface and total GluA1 demonstrated that over-expression of FRRS1L did not change surface levels, total levels, or surface to total ratio of GluA1 expression (**Figure 4A**), suggesting that increased expression of FRRS1L is not sufficient to alter AMPAR trafficking to the neuronal surface.

To corroborate the immunocytochemical data, we performed electrophysiological recordings to measure AMPAR-mediated synaptic transmission in neurons over-expressing FRRS1L. Toward this end, we biolistically transfected cultured organotypic hippocampal slices with gold particles that were coated with plasmids expressing FRRS1L-IRES-EGFP. Two to five days after transfection, we performed simultaneous dual whole-cell voltage clamp recordings (dual recordings) that were made from a transfected pyramidal cell (identified by EGFP) and a neighboring EGFP-negative neuron to detect AMPAR- and NMDA receptor (NMDAR)-mediated EPSCs. A stimulating electrode was placed at the Schaffer collateral pathway to evoke EPSCs in both cells. We found that over-expression of FRRS1L did not change either AMPA or NMDA EPSCs (**Figures 4B,C**), and the weighted decay time constant of the AMPA EPSC (**Figure 4D**). In addition, the PPR, a measure of presynaptic neurotransmitter release probability, of AMPA EPSCs in the postsynaptic neurons over-expressing FRRS1L was not changed (**Figure 4E**). These data indicate that over-expression of FRRS1L in hippocampal pyramidal neurons does not affect the strength of excitatory synaptic transmission.

### Genetic Deletion of FRRS1L Significantly Reduces Surface AMPARs in Neurons

**FIGURE 5:**
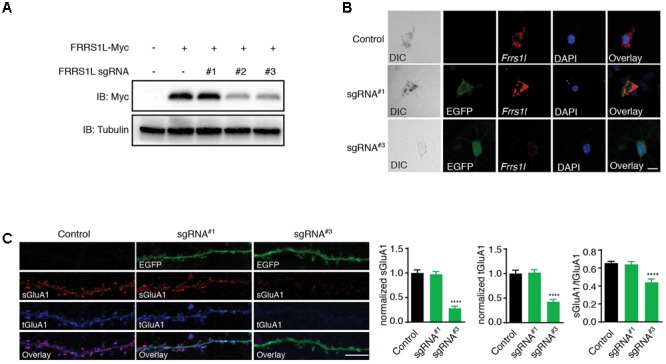
Genetic deletion of FRRS1L reduces the surface and total expression levels of GluA1 in cultured hippocampal neurons. **(A)** Screening candidate sgRNAs against FRRS1L in HEK cells. Western blotting analysis show that sgRNA^#2^ and sgRNA^#3^, but not sgRNA^#1^, strongly reduced FRRS1L-Myc expression in HEK cells, *N* = 3 independent repeats. Uncropped scans of Western blots were shown in Supplementary Figure 3. **(B)** *In situ* hybridization (ISH) data show that sgRNA^#3^, but not sgRNA^#1^, eliminated *Frrs1l* mRNA expression (in red) in cultured hippocampal neurons (Control: score 3, *n* = 20; sgRNA^#1^: score 3, *n* = 15; sgRNA^#3^: score 0–1, *n* = 18). Scale bar, 10 μm. **(C)** sgRNA^#3^ induced genetic deletion of FRRS1L impaired sGluA1 and tGluA1 expressions in cultured hippocampal neurons. Representative dendrite images from a non-transfected control neuron or neurons expressing FRRS1L sgRNAs (Control: *n* = 11; sgRNA^#1^: *n* = 14; sgRNA^#3^: *n* = 13; normalized sGluA1: F (2,38) = 12.58, *p* < 0.0001; normalized tGluA1: F (2,38) = 16.74, *p* < 0.0001, sGluA1/tGluA1: F (2,38) = 9.27, *p* < 0.0001; one-way ANOVA with *post hoc* Fisher’s LSD test) are shown in the left panels. Scale bar, 20 μm.

To further determine the physiological role of FRRS1L protein in neurons, we utilized the CRISPR-Cas9 system to develop single-guide RNAs (sgRNAs) to target *Frrs1l* gene loci in mouse genome. Among three FRRS1L sgRNA candidates, sgRNA candidate 1 (sgRNA^#1^) did not decrease the expression of co-transfected FRRS1L-Myc in HEK cells, while the other two candidates (sgRNA^#2^ and sgRNA^#3^) effectively reduced the expression levels of FRRS1L-Myc (**Figure 5A**). We further tested the effectiveness of sgRNA^#1^ and sgRNA^#3^ in inactivating the *Frrs1l* gene in neurons (sgRNA vectors simultaneously expressed EGFP for cell identification) with an *in situ* hybridization assay to measure FRRS1L mRNAs in dissociated hippocampal primary cultures (Commercially available FRRS1L antibodies were not suitable for immunolabeling of endogenous FRRS1L in neurons, and thus we could not perform immunocytochemical experiments in neurons). We found that in neurons expressing sgRNA^#3^, FRRS1L mRNAs were essentially not detectable as compared to FRRS1L mRNAs in non-transfected neurons (Control) or sgRNA^#1^-expressing neurons (**Figure 5B**), indicating that we had successfully achieved single-cell KO of FRRS1L in neurons expressing sgRNA^#3^.

To study the role of FRRS1L in AMPAR trafficking to the plasma membrane in neurons, we transfected sgRNA^#3^, or sgRNA^#1^ as a control, at DIV2 (days *in vitro*) neuronal cultures and measured surface and total GluA1 expression levels at DIV16. We found that surface levels of GluA1 were significantly reduced in neurons expressing sgRNA^#3^ (**Figure 5C**). Interestingly, total GluA1 expression levels were also reduced in sgRNA^#3^-expressing neurons (**Figure 5C**), suggesting that FRRS1L is critical for the stability of GluA1 protein in neurons. Furthermore, the surface to total ratio of GluA1 expression was significantly decreased in neurons expressing sgRNA^#3^ (**Figure 5C**), indicating that targeting of GluA1 to the neuronal surface is impaired in neurons lacking FRRS1L. In contrast, transfection of sgRNA^#1^ into neurons changed neither surface nor total GluA1 (**Figure 5C**). Taken together, these data demonstrate that FRRS1L is critical for GluA1 stability in neurons and also plays a role in GluA1 trafficking to the neuronal surface.

### FRRS1L Is Important for AMPAR-Mediated Synaptic Transmission *in Vitro* and *in Vivo*

**FIGURE 6:**
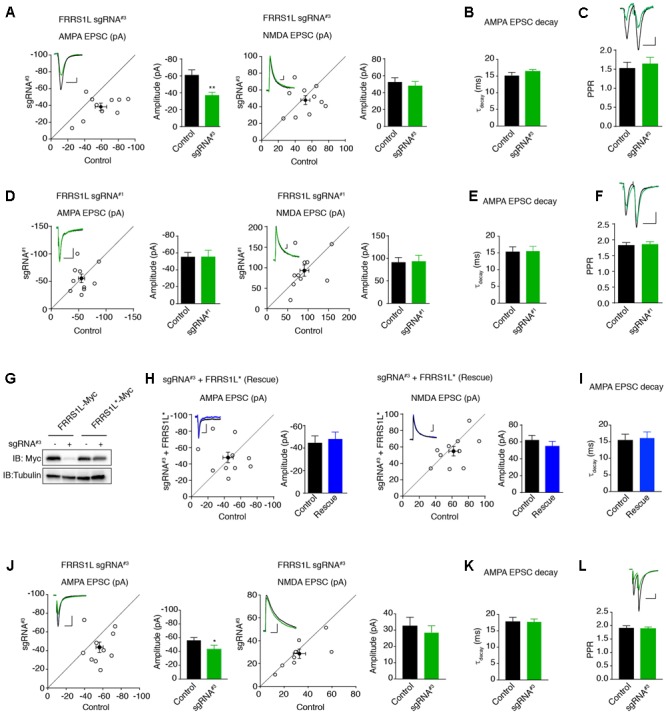
Single-cell genetic deletion of FRRS1L *in vitro* and *in vivo* significantly reduces AMPAR-mediated synaptic transmission. **(A)** Scatter plot of AMPA and NMDA EPSCs in pyramidal neurons expressing sgRNA^#3^ and nearby control neurons in cultured organotypic hippocampal slices. Expression of sgRNA^#3^ significantly reduced AMPA EPSCs (*n* = 10, *p* < 0.01, paired *t*-test), without affecting NMDA EPSCs (*n* = 10, *p* = 0.56, paired *t*-test). Scale bar, 20 pA and 100 ms. **(B)** The weighted decay time constant was not changed in neurons expressing sgRNA^#3^ (*n* = 10, *p* = 0.25, *t*-test). **(C)** There was no change of PPR in neurons expressing sgRNA^#3^ (*n* = 11, *p* = 0.36, *t*-test). Scale bar, 20 pA and 100 ms. **(D)** Scatter plot of AMPA and NMDA EPSCs in pyramidal neurons expressing sgRNA^#1^ and nearby control neurons in cultured organotypic hippocampal slices. Expression of sgRNA^#1^ did not change AMPA (*n* = 10, *p* = 0.98, paired *t*-test) and NMDA (*n* = 10, *p* = 0.88, paired *t*-test) EPSCs. Each open circle represents one paired recording in the scatter plots, and the solid black point is the average of all pair recordings. Scale bar, 20 pA and 100 ms. **(E)** The weighted decay time constant was not changed in neurons expressing sgRNA^#1^ (*n* = 10, *p* = 0.97, *t*-test). **(F)** There was no change of PPR in neurons expressing sgRNA^#1^ (*n* = 10, *p* = 0.76, *t*-test). Scale bar, 20 pA and 100 ms. **(G)** sgRNA^#3^ failed to reduce the expression of sgRNA resistant FRRS1L (FRRS1L^∗^) in HEK cells. Western blotting analysis showed that sgRNA^#3^ strongly reduced WT FRRS1L-Myc, but not FRRS1L-Myc^∗^, expression in HEK cells. Uncropped scans of Western blots were shown in Supplementary Figure 3. **(H)** Scatter plot of AMPA and NMDA EPSCs in pyramidal neurons expressing sgRNA^#3^ together with FRRS1L^∗^ and nearby control neurons in cultured organotypic hippocampal slices. Expression of FRRS1L^∗^ rescued the AMPA EPSC deficit induced by sgRNA^#3^ (AMPA, *n* = 10, *p* = 0.74; NMDA, *n* = 10, *p* = 0.24; paired *t*-test). Scale bar, 20 pA and 100 ms. **(I)** The weighted decay time constant was not changed in neurons expressing sgRNA^#3^ together with FRRS1L^∗^ (*n* = 10, *p* = 0.85, *t*-test). **(J)** Scatter plot of AMPA and NMDA EPSCs in CA1 neurons expressing sgRNA^#3^ and nearby control neurons in acute hippocampal slices from p14-16 mice that were electroporated *in utero* at E14.5-15.5. Expression of sgRNA^#3^ *in vivo* significantly reduced AMPA EPSCs (*p* < 0.05, *n* = 9, paired *t*-test with Wilcoxon test), without affecting NMDA EPSCs (*n* = 9, *p* = 0.53, paired *t*-test). Scale bar, 20 pA and 100 ms. **(K)** The weighted decay time constant was not changed in neurons expressing sgRNA^#3^ (*n* = 9, *p* = 0.88, *t*-test). **(L)** sgRNA^#3^ did not change the PPR (control and sgRNA^#3^: *n* = 9, *p* = 0.81, *t*-test). Scale bar, 20 pA and 100 ms.

Finally, we examined the effect of single-cell KO of FRRS1L on AMPAR-mediated synaptic transmission. We first biolistically transfected cultured organotypic hippocampal slices with sgRNA^#3^ or sgRNA^#1^ plasmids. Approximately 2 weeks after transfection, we performed dual recordings to measure glutamatergic synaptic transmission in pyramidal neurons. Expression of sgRNA^#3^ specifically reduced the amplitude of AMPA EPSCs by ∼30%, without affecting NMDA EPSC amplitudes (**Figure 6A**). In addition, there was no change of PPR (**Figure 6C**), suggesting that loss of FRRS1L in the postsynaptic neurons does not alter presynaptic neurotransmitter release probability. In contrast, expression of sgRNA^#1^ did not affect AMPA and NMDA EPSCs nor PPR (**Figures 6D,F**). Importantly, an sgRNA^#3^-resistant FRRS1L mutant (FRRS1L^∗^) fully rescued the deficit of AMPA EPSCs in pyramidal neurons expressing sgRNA^#3^ (**Figures 6G,H**), demonstrating that the effect of sgRNA^#3^ on AMPA EPSCs is due to the loss of FRRS1L protein. Furthermore, none of the manipulations changed the weighted decay time constants of AMPA EPSCs (**Figures 6B,E,I**). Taken together, these results show that FRRS1L is critical for AMPAR-mediated synaptic transmission in hippocampal pyramidal neurons.

To study the role of FRRS1L in the regulation of glutamatergic synaptic transmission *in vivo*, we electroporated plasmids *in utero* to sparsely express sgRNA^#3^ in hippocampal progenitor cells in E14.5 mice embryos to inactivate *Frrs1l* alleles and then performed dual recordings to measure glutamatergic synaptic transmission in acute hippocampal slices prepared from 2- to 3-week-old mice. We found that single-cell KO of FRRS1L with sgRNA^#3^ in CA1 pyramidal neurons in acute hippocampal slices significantly decreased AMPA, but not NMDA, EPSCs (**Figure 6J**). In addition, there was no change of the weighted decay time constant of AMPA EPSCs and PPR (**Figures 6K,L**). Thus, FRRS1L is important for AMPAR-mediated synaptic transmission *in vivo*.

## Discussion

Recent proteomic screenings have identified a growing number of membrane proteins that are associated with native AMPAR complexes in the brain (Schwenk et al., 2009, 2012; von Engelhardt et al., 2010; Chen et al., 2014; Bettler and Fakler, 2017). Subsequent functional investigations have revealed that many of these membrane proteins play critical roles in the regulation of trafficking and/or function of AMPARs (Schwenk et al., 2009, 2012; Kato et al., 2010; Shi et al., 2010; von Engelhardt et al., 2010; Gill et al., 2011, 2012; Coombs et al., 2012; Herring et al., 2013; Boudkkazi et al., 2014; Gratacos-Batlle et al., 2014; Fado et al., 2015; McGee et al., 2015; Erlenhardt et al., 2016; Gu et al., 2016a; Klaassen et al., 2016; Wei et al., 2016; Mao et al., 2017). In this study, we have focused on FRRS1L, a protein isolated in AMPAR complexes in the brain, but its function in the regulation of AMPAR-mediated synaptic transmission was unclear. Underscoring the importance of FRRS1L in neuronal function, recent human genetic studies have shown that loss-of-function mutations of FRRS1L in human lead to severe impairments of motor and cognitive functions (Madeo et al., 2016; Shaheen et al., 2016).

Immunocytochemical data in neuronal cultures show that only a minor fraction of immunolabeling of expressed FRRS1L co-localizes with the excitatory synaptic marker, PSD-95, indicating that FRRS1L is largely localized at non-synaptic subcellular regions in neuronal dendrites. Moreover, there is a substantial co-localization between expressed FRRS1L and endogenous AMPAR subunit GluA1, suggesting that FRRS1L associates with a subpopulation of AMPARs. Interestingly, our data demonstrate that FRRS1L, but not TARP γ8 or CNIH2, associates with dynein, but not kinesin KIF5B, vesicles. Previous studies have shown that microtubule-based motors, kinesin and dynein, are important for the long-range dendritic transport of vesicles containing the assembled AMPARs out of the ER and other compartments of the endomembrane system, and for the regulation of excitatory synaptic transmission (Kim and Lisman, 2001; Setou et al., 2002; Kapitein et al., 2010; Hoerndli et al., 2013). The association of FRRS1L with dynein vesicles suggests that FRRS1L-containing AMPARs could be transported by dynein-dependent mechanisms. However, the exact role of FRRS1L in dynein-based AMPAR trafficking remains to be examined.

In heterologous HEK cells, co-expression of FRRS1L with the AMPAR subunit GluA1 or GluA2 does not change the surface abundance of the receptor subunits. Similarly, expression of AMPAR subunits together with FRRS1L does not alter 1 mM glutamate-evoked whole-cell currents in the presence of cyclothiazide in HEK cells. Thus, FRRS1L on its own appears not be sufficient to change the expression of AMPARs at the plasma membrane, which is different from other AMPAR auxiliary subunits that often either promote or diminish AMPARs at the cell surface in heterologous cells (Tomita et al., 2004; Schwenk et al., 2009; von Engelhardt et al., 2010; Shanks et al., 2012; Gratacos-Batlle et al., 2014; Gu et al., 2016a; Erlenhardt et al., 2016; Wei et al., 2016). Furthermore, in neurons, over-expression of FRRS1L changes neither the surface GluA1 nor AMPAR-mediated synaptic transmission. These data indicate that increased expression of FRRS1L in neurons is not sufficient to alter AMPAR trafficking to the plasma membrane and FRRS1L is unlikely a limiting factor in determining the expression levels of AMPARs at the surface of cultured neurons in our experiments. Our findings that over-expression of FRRS1L does not change excitatory synaptic transmission and that single-cell genetic deletion of FRRS1L decreases AMPAR-mediated EPSCs resemble previous work on CNIH2. In hippocampal CA1 neurons, it has been reported that over-expression of CNIH2 in hippocampal neurons does not change excitatory synaptic transmission (Herring et al., 2013). However, genetic deletion of CNIH2 in hippocampal CA1 neurons strongly diminishes AMPAR-mediated synaptic transmission (Herring et al., 2013). In addition, a recent study has shown that in HEK cells, FRRS1L (C9orf4) can reduce non-saturating 100 μM glutamate-evoked, GluA1 homomer-mediated calcium influx, but increase GluA1-TARP γ8 complex-mediated calcium influx (Erlenhardt et al., 2016). These data suggest that while FRRS1L does not change the number of receptors at the cell surface as shown in our study, it can modulate the steady-state currents in the absence of cyclothiazide and can functionally modulate AMPAR-TARP γ8 complexes in heterologous cells.

sgRNA-based single-cell KO of FRRS1L shows that FRRS1L plays an important role in the regulation of AMPAR-mediated synaptic transmission in hippocampal pyramidal neurons. Indeed, FRRS1L KO in hippocampal neurons both *in vitro* and *in vivo* reduces the AMPA EPSCs. Importantly, a sgRNA resistant FRRS1L fully rescues the AMPA EPSC deficit in the sgRNA-positive neurons. Similarly, FRRS1L KO in neuronal cultures significantly reduces GluA1 staining on the neuronal surface. These data are in agreement with a previous study in a neuronally differentiated cell line in which knockdown of FRRS1L diminished AMPA-induced whole-cell currents (Madeo et al., 2016). In addition, GluA1 total protein levels are reduced in FRRS1L KO neurons, suggesting that FRRS1L is important for GluA1 stability and/or biogenesis. Furthermore, the surface to total ratio of GluA1 expression is significantly decreased in neurons lacking FRRS1L. Thus, it is likely that decreased surface levels of GluA1 in FRRS1L KO neurons are caused by a combined effect of reduced total GluA1 expression and impaired delivery of GluA1 to the neuronal surface. It has been reported that expression levels of AMPAR receptor subunits are reduced in neurons lacking other AMPAR auxiliary subunits, including TARPs, CNIHs, PORCN or CKAMP44 (Rouach et al., 2005; Herring et al., 2013; Khodosevich et al., 2014; Erlenhardt et al., 2016), indicating that AMPAR auxiliary subunits commonly play important roles in the regulation of AMPAR stability in neurons.

While this manuscript was in preparation, an elegant study on FRRS1L that reveals a critical role of FRRS1L in early biogenesis of AMPARs and brain function was published (Brechet et al., 2017). This work shows that endogenous FRRS1L is largely confined in the ER, is important for AMPAR biogenesis and is required for AMPAR-mediated synaptic transmission (Brechet et al., 2017). Our data support these conclusions (Brechet et al., 2017). Indeed, similar to our single-cell KO data, it was reported that shRNA-mediated knockdown of FRRS1L diminished AMPAR-medicated synaptic transmission in hippocampal neurons (Brechet et al., 2017). In addition, our data show the reduced expression levels of GluA1 in FRRS1L KO neurons (**Figure 5C**), which is consistent with a role of FRRS1L in AMPAR biogenesis (Brechet et al., 2017). We also found that over-expression or single-cell KO of FRRS1L in hippocampal neurons did not change the AMPA EPSC decay kinetics, which is in agreement with the recent report (Brechet et al., 2017). Furthermore, our data show that FRRS1L, but not TARP γ8 or CNIH2, associates with dynein, indicating that FRRS1L-containing AMPARs represent a distinct receptor population, consistent with the reported proteomic analysis of the unique FRRS1L-AMPAR assemblies (Brechet et al., 2017). It has also been reported that over-expression of FRRS1L increases AMPAR-mediated synaptic transmission (Brechet et al., 2017), which is different from our findings that FRRS1L over-expression did not change AMPA EPSCs. This discrepancy can be explained by different over-expression techniques (viral transduction *in vivo* versus plasmid transfection *in vitro* in our study), different experimental preparations (acute rat hippocampal slices versus mouse hippocampal organotypic cultures in our study), different neuronal types for recording (hippocampal mossy cells, interneurons or CA3 neurons versus hippocampal CA1 neurons in our study) and different over-expression durations (∼ 2 weeks versus 2–5 days in our study). It is possible that over-expression of FRRS1L in neurons may not represent the appropriate approach to study the FRRS1L function in synaptic transmission, as over-expressed FRRS1L can reach the neuronal surface (**Figure 1F**), but the vast majority of native FRRS1L do not (Brechet et al., 2017). Nevertheless, our findings of FRRS1L localization at dynein-positive vesicles together with the published report (Brechet et al., 2017) suggest that FRRS1L may primarily regulate the strength of AMPAR-mediated synaptic transmission through the modulation of AMPAR biogenesis and/or transport at the early secretory pathways.

In summary, we have provided some basic characterizations of FRRS1L in heterologous cells and hippocampal neurons. Our findings reveal an important role of FRRS1L in the regulation of excitatory synaptic transmission. Future work examining the molecular mechanisms underlying the regulation of AMPARs by FRRS1L and the potential role of dynein in the transport of FRRS1L-containing AMPARs will contribute to our understanding of AMPAR biogenesis and trafficking.

## Author Contributions

WH, HW, JL, SZ, and WL designed the research. HW performed immunocytochemical experiments in neurons, and electrophysiological recordings in HEK cells and hippocampal slice cultures. WH performed vesicle immunoisolation, immunocytochemical experiments in neuronal cultures and dual whole-cell current recording in acute hippocampal slices. WH and HW performed biochemical characterization. JL performed immunostaining in HEK cells. WL and WH wrote the paper. All authors read and commented on the manuscript, and approved the final version of the manuscript.

## Conflict of Interest Statement

The authors declare that the research was conducted in the absence of any commercial or financial relationships that could be construed as a potential conflict of interest.

## Abbreviations

ABHD6: α/β-Hydrolase domain-containing 6
AMPA: a-amino-3-hydroxy-5-methyl-4-isoxazolepropionic acid
ANOVA: analysis of variance
CKAMP: cysteine-knot AMPAR modulating protein
CNIHs: cornichons
CRISPR: clustered regularly interspaced short palindromic repeats
DIV: days *in vitro*
EDTA: ethylenediaminetetraacetic acid
EGFP: enhanced GFP
EPSC: excitatory postsynaptic currents
FRRS1L: Ferric Chelate Reductase 1 Like protein
GPI: glycophosphatidylinositol
GSG1L: germ cell-specific gene 1-like protein
HA: hemagglutinin
HEK: human embryonic kidney
IP: immunoprecipitation
KO: knock out
NMDA: *N*-methyl-D-aspartate
PNS: post-nuclear supernatant
PNSM: post-nuclear supernatant membrane fractions
PORCN: porcupine
PPR: paired pulse ratio
PSD: postsynaptic density
sgRNA: single-guide RNA
TARP: transmembrane AMPA receptor regulatory proteins
vGluT: vesicular glutamate transporter 1
WT: wild-type
