# Supplementary material for: Ferric Chelate Reductase 1 Like Protein (FRRS1L) Associates with Dynein Vesicles and Regulates Glutamatergic Synaptic Transmission
Source: Front Mol Neurosci. 2017 Dec 8;10:402. doi: 10.3389/fnmol.2017.00402 (PMC5727121; doi:10.3389/fnmol.2017.00402)
Supplement: Supplementary file 1 [file Data_Sheet_1.PDF]

Figure.1

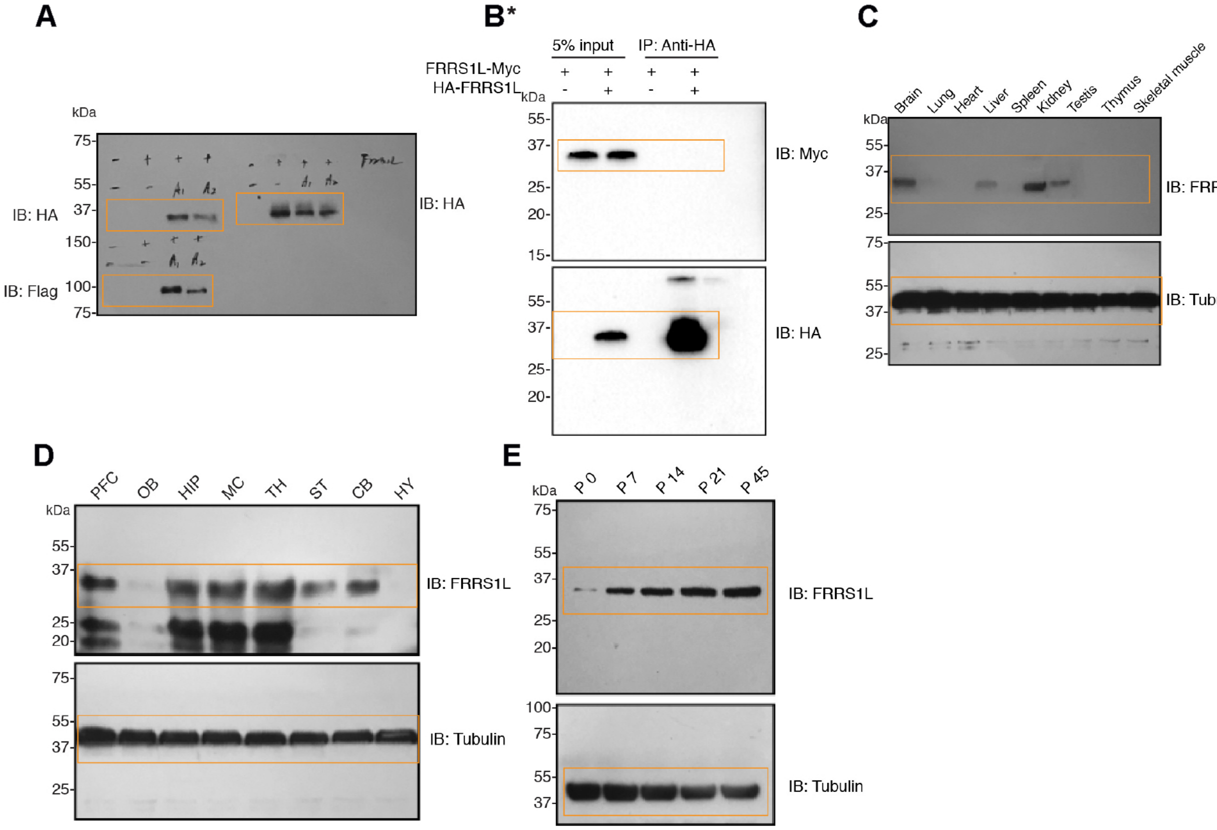

Supplementary Fig.1. Uncropped scans of Western blots shown in Fig.1**A-E**. Orange rectangles labelled the cropped regions. *Images were originally scanned with a commercial imager. Other images were scanned from films.

Figure.2

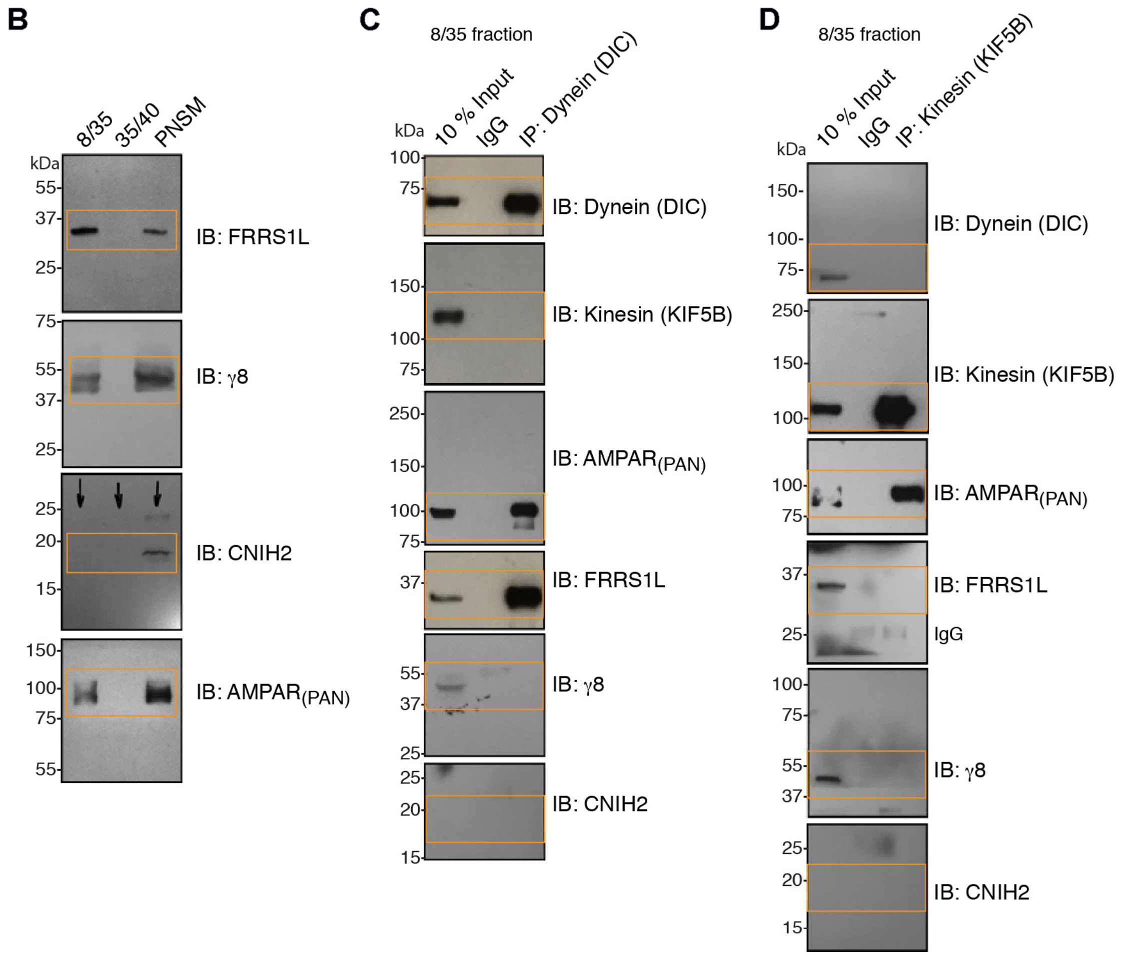

Supplementary Fig.2. Uncropped scans of Western blots shown in Fig.2**B-D**. Orange rectangles labelled the cropped regions.

Figure.5A*

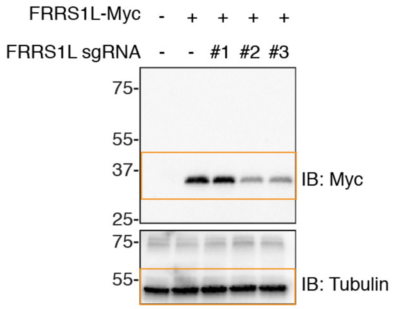

Figure.6G*

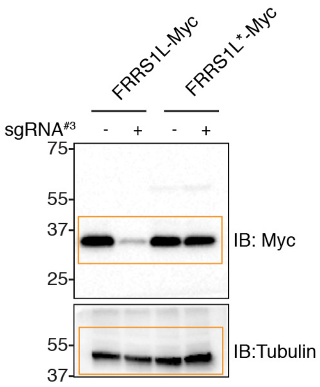

Supplementary Fig.3 Uncropped scans of Western blots shown in Fig.5A and Fig.6G. Orange rectangles labelled the cropped regions. *Images were originally scanned with a commercial imager.
